# The Hippo pathway in endometrial cancer: a potential therapeutic target?

**DOI:** 10.3389/fonc.2023.1273345

**Published:** 2023-10-20

**Authors:** Xinyun Shen, Qianqian Li, Yiqing Sun, Lingli Chen, Fengxia Xue, Wenyan Tian, Yingmei Wang

**Affiliations:** ^1^Department of Gynecology and Obstetrics, Tianjin Medical University General Hospital, Tianjin, China; ^2^Tianjin Key Laboratory of Female Reproductive Health and Eugenics, Tianjin Medical University General Hospital, Tianjin, China

**Keywords:** hippo pathway, endometrial cancer (EC), tumor microenvironment, drug resistance, treatment

## Abstract

Endometrial cancer, one of the most prevalent malignant cancers tumors of the female reproductive tract, has been increasing in incidence and mortality rates around the world. The Hippo pathway, one of the eight traditional human cancer signaling pathways, is an intricate signaling network that regulates cell proliferation, differentiation, and migration as well as restricting organ size in response to a range of intracellular and extracellular signals. Inhibiting the Hippo pathway results in aberrant activation of its downstream core component YAP/TAZ, which can enhance cancer cells’ metabolism and maintain their stemness. Additionally, the Hippo pathway can modulate the tumor microenvironment and induce drug resistance, where tumorigenesis and tumor progression occur. However, the Hippo pathway has been little researched in endometrial cancer. Here, we aim to review how the Hippo pathway contributes to the onset, development and the potential treatment of endometrial cancer with the aim of providing new therapeutic targets.

## 1 Introduction

Endometrial cancer (EC) is one of the most common malignant tumors of the female reproductive tract and the sixth most common cancer in women. Globally, EC prevalence is second only to cervical cancer and both its incidence and fatality rate have been rising. According to the latest statistics from the American Cancer Society, the 5-year survival rate of EC patients with distant metastases is only 18% (1). Immune checkpoint inhibitors have been used to treat advanced and metastatic EC with some success, but tolerance is a challenge affecting two-thirds of patients with grade 3-4 treatment-related toxicity (2). Therefore, there is an urgent need to explore further therapeutic targets to achieve new advances. The Hippo signaling pathway is one of the eight traditional human cancer signaling pathways and its function in malignancy has drawn considerable attention. An increasing amount of research has shown that it is strongly linked to tumorigenesis, invasion, recurrence, metastasis, and drug resistance. In this article, we review the Hippo pathway and its role in the tumorigenesis, drug resistance and therapeutic targets in endometrial cancer.

## 2 Hippo signaling pathway

The Hippo pathway, named for the hippo-like phenotype of its main protein kinase, consists of a collection of kinases that were discovered in Drosophila, but which is highly conserved across species. Furthermore, this cascade of kinases has also been demonstrated in mammalian cells (3). The Yes-associated protein (YAP) and the transcriptional co-activator with PDZ binding motif (TAZ) are two crucial co-activators of transcription whose activity is inhibited by activation of the Hippo pathway. When the Hippo pathway is turned on, mammalian sterile 20-like kinases 1 (MST1/2) are activated and phosphorylate large tumor suppressor 1/2 (LATS1/2) kinases to activate them; facilitated by adaptors salvador homolog-1 (SAV1) and Mps one binder kinase activator 1A/1B (MOB1A/B). Activated LATS1/2 phosphorylate YAP/TAZ, negatively regulating its translocation from the cytoplasm to the nucleus. Conversely, when the Hippo pathway is inhibited, activation of YAP/TAZ takes place leading to translocation from the cytoplasm to the nucleus acting on DNA after binding to the transcription-enhancing association domain (TEAD) family, which in turn activates TEAD-mediated gene transcription. In brief, MST1/2-LATS1/2-YAP/TAZ-TEAD constitute the core components of the mammalian Hippo pathway, and YAP/TAZ serves as its primary effector.

The Hippo pathway can be activated in response to a variety of intracellular and extracellular signals, including intercellular contact, cell polarity, energy stress, cytoskeleton and G protein-coupled receptor (GPCR) signals (4). Among these, GPCR signals both activate and inhibit the Hippo-YAP pathway: the direction depending on the coupled G protein. Serum-borne lysophosphatidic acid and sphingosine 1-phosphophate act through G12/13-coupled receptors to inhibit the LATS1/2, thereby activating YAP/TAZ. Adrenergic β2 receptors, dopamine D1 receptors, and glucagon receptors, by contrast, activate GS signal inducing YAP/TAZ phosphorylation (5).

**Figure 1:**
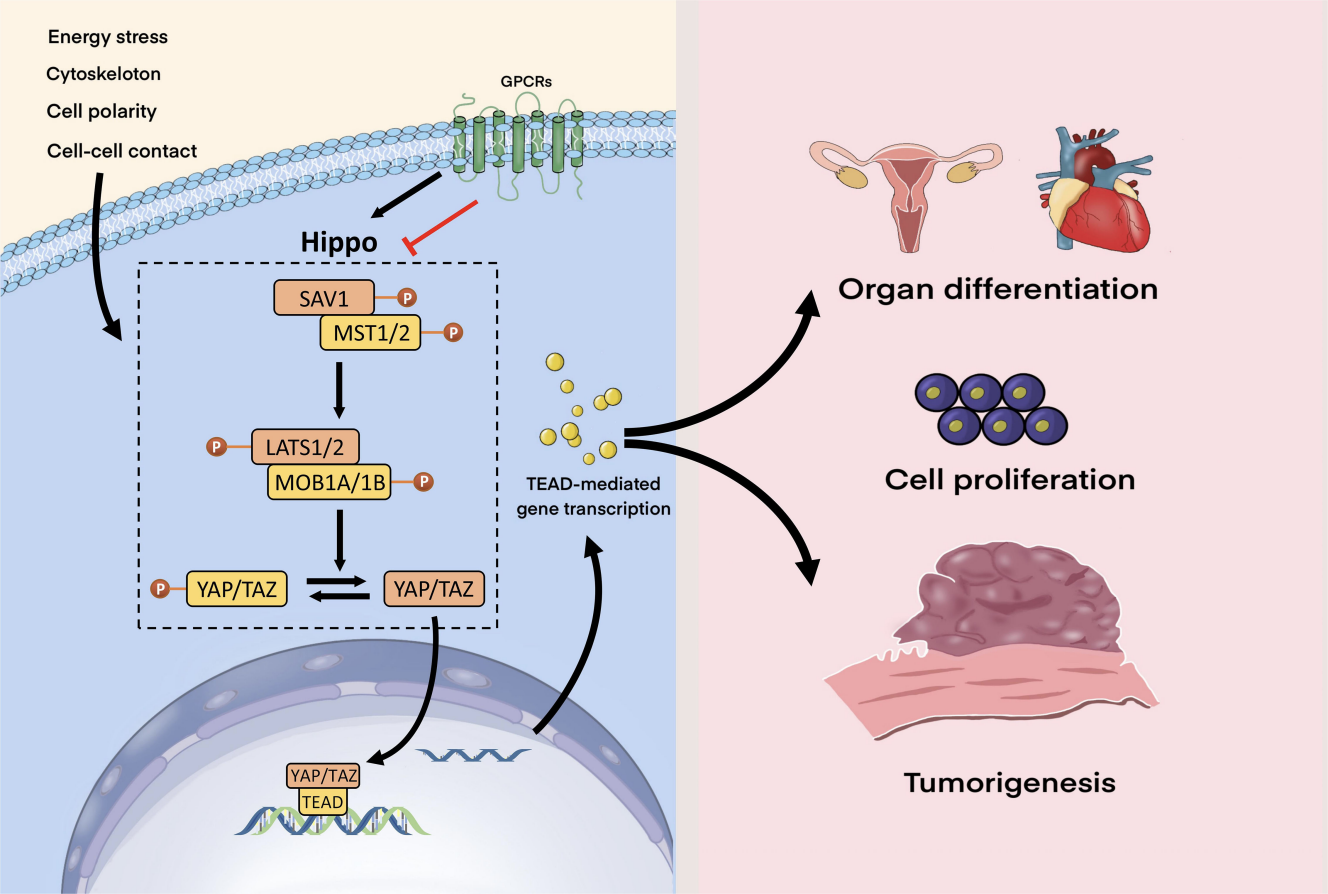
Hippo signaling pathway. The Hippo pathway can respond to a wide range of signals, including those from the cytoskeleton, GPCRs, energy stress, intercellular contact, and cell polarity. GPCR signals can both activate and inhibit the Hippo-YAP pathway. When Hippo is turned on, activated the MST1/2-SAV1 complex phosphorylates the LATS1/2-MOB1A/B complex in the Hippo kinase cascade, which then phosphorylates YAP/TAZ. When the Hippo pathway is suppressed, YAP/TAZ is activated, translocates to the nucleus, binds to TEAD, and thereby stimulates TEAD-mediated gene transcription. The Hippo pathway regulates organ development, cell proliferation, and growth under normal circumstances, but when it malfunctions, tumorigenesis may occur.

In general, the Hippo pathway finely regulates cell growth, proliferation and organ differentiation in response to various signals. However, when the Hippo pathway is dysfunctional, its downstream components are inhibited and YAP/TAZ is abnormally activated, which may lead to tumorigenesis (**Figure 1**).

The Hippo pathway can respond to a wide range of signals, including those from the cytoskeleton, GPCRs, energy stress, intercellular contact, and cell polarity. GPCR signals can both activate and inhibit the Hippo-YAP pathway. When Hippo is turned on, activated the MST1/2-SAV1 complex phosphorylates the LATS1/2-MOB1A/B complex in the Hippo kinase cascade, which then phosphorylates YAP/TAZ. When the Hippo pathway is suppressed, YAP/TAZ is activated, translocates to the nucleus, binds to TEAD, and thereby stimulates TEAD-mediated gene transcription. The Hippo pathway regulates organ development, cell proliferation, and growth under normal circumstances, but when it malfunctions, tumorigenesis may occur.

## 3 Hippo pathway and cancer

The Hippo pathway is one of the eight traditional human cancer signaling pathways that was identified by The Cancer Genome Atlas after analysis of more than 9,000 cancers (6). As major effectors of the Hippo pathway, activation and epigenetic modification of YAP/TAZ is widespread in a variety of human cancers, where YAP/TAZ is essential for cancer initiation, progression and metastasis (7). The upstream regulation of Hippo, the oncogenic impacts of YAP/TAZ activation and the effects on the tumor microenvironment and drug resistance are summarized in the section that follows.

### 3.1 Upstream regulation of the Hippo pathway

In contrast to numerous prevalent cancer signal pathways, the Hippo pathway is characterized by comparatively few mutations in its upstream genes, which is therefore a challenging field to explore. The upstream regulation of Hippo by other tumor-associated pathways represents an important starting point for research.

#### 3.1.1 Phosphatidylinositol 3-kinase signaling pathway

The PI3K signaling pathway plays a pivotal role in cancer and emerging evidence suggests that it interacts with the Hippo pathway to drive cell proliferation and malignancy. YAP/TAZ has been demonstrated to be a crucial mediator of PI3K pathway-induced mammary tumors and plays a synergistic role with the PI3K pathway in mammary cell transformation. The catalytic subunits of PI3K, PIK3CA and PIK3CB, activate YAP/TAZ through PI3K-PDK1-AKT signaling, with AKT positively regulates the expression of YAP, thereby promoting tumorigenesis and inhibiting cell apoptosis (8). In addition, in patients with hepatocellular carcinoma, deficiency of phosphatase and tensin homolog (PTEN) activated AKT, which activated YAP/TAZ, which in turn induced transcription of insulin receptor substrate 2 (IRS2). Upregulation of IRS2 enhanced PI3K-AKT activation, and further stimulated TAZ. This positive feedback loop eventually leads to the development of a fatty liver or malignant transformation (9). These studies suggest that the PI3K pathway can act on the Hippo pathway and thus promote tumorigenesis.

#### 3.1.2 Mitogen-activated protein kinase signaling pathway

In addition to the PI3K pathway, the MAPK signaling pathway is also involved in regulating the Hippo pathway. p38MAPK links the stress response to the Hippo signaling pathway. p38 promotes the cytoplasmic translocation of TEAD and inhibits the growth of YAP-driven cancer cells under various cellular stress stimuli. Furthermore, stress caused by cell division failure can activate LATS2, thereby inactivating YAP/TAZ (10). MAP4K1/2/3/4/6/7 can phosphorylate and activate LATS1/2 in parallel with MST1/2, in turn inactivating downstream YAP/TAZ phosphorylation. In EC, Striatin-4 (STRN4), a component of the striatin-interacting phosphatase and kinase complex exerts oncogenic effects by promoting the activation of YAP, a process that requires the involvement of MAP4K binding to STRN4 with inhibition of its ability to phosphorylate YAP/TAZ (11). Dysregulation of the Hippo pathway was also essential for schwannomagenesis where MAPK signaling acted as a modifier for schwannoma formation. Co-targeting YAP/TAZ and MAPK signaling demonstrated a synergistic therapeutic effect on schwannomas (12).

#### 3.1.3 AMP-activated protein kinase

The impact of AMPK on the Hippo pathway has been demonstrated in a variety of studies. In response to energy stress, AMPK, a cellular energy sensor, directly phosphorylated YAP at the S94 location or indirectly activates the LATS kinase via AMOTL1 or Rho GTPase, limiting YAP binding to TEAD and inhibiting cell growth (13). Metformin is a first-line diabetes medication with anticancer efficacy, which can induce AMPK to directly phosphorylate YAP and reduce YAP activity, hence suppressing tumorigenesis. This effect was especially evident in tumors with high YAP activity (14). Since Hippo itself is regulated by energy signaling, energy stress can activate the AMPK and Hippo pathways in parallel; both enhancing YAP phosphorylation.

#### 3.1.4 Non-coding RNAs

NcRNAs, including microRNAs (miRNAs), circular RNAs (circRNAs), and long non-coding RNAs (lncRNAs), have also been closely involved in the modulation of Hippo pathways in addition to the three signaling routes mentioned above. For example, pancreatic cancer (PC) cells have much higher levels of miR-10a expression than adjacent normal pancreatic cells. It promoted the epithelial-mesenchymal transition (EMT) and stemness of PC by inhibiting WW and C2 domain containing 2 expression via activation of YAP in the Hippo pathway (15). In addition to miRNAs, an enhanced level of the circRNA circPPP1R12A has been discovered in the cytoplasm of colorectal cancer cells. Furthermore, the circRNA circPPP1R12A encodedthe conserved 73-aa short peptide PPP1R12A-C, which increased the growth of colorectal cancer cells. Stimulating the Hippo-YAP, circPPP1R12A-73aa enhanced the ability of CC cells to proliferate, migrate, and invade (16). lncRNAs can also target core components of the Hippo pathway. In breast cancer, the ROR1-HER3 pathway recruited the lncRNA MAYA and the methyltransferase NSUN6, driving MST1 methylation and consequently reducing MST1 kinase activity. This resulted in LATS1 dephosphorylation and YAP activation and accumulation in the nucleus, which stimulated the expression of target genes associated with breast cancer tumor cell proliferation and bone metastasis, ultimately causing osteoclast differentiation and bone resorption (17).

#### 3.1.5 Neurofibromatosis type 2/Merlin

Merlin is encoded by the NF2 gene, performing critical roles in inhibiting cell growth and proliferation as an upstream activator of the Hippo pathway (18). In melanoma, mesothelioma, and meningiomas, Merlin is thought to act as a tumor suppressor by activating Hippo (19–21). By recruiting LATS1/2 directly to membranes, Merlin can initiate the Hippo signaling and phosphorylate the downstream effectors, inactivating YAP/TAZ and preventing their translocation to the nucleus and inhibiting their function as transcription co-activators (22). Studies have revealed that Merlin controls Hippo signaling by inhibiting the CRL4^DCAF1^ E3 ubiquitin ligase in the nucleus. Loss of Merlin’s inhibition of CRL4^DCAF1^ stabilizes LATS 1/2 and hence activates YAP, which is essential for triggering YAP-driven transcription and oncogenesis (23, 24).

### 3.2 Cancer progressive effects of YAP/TAZ activation

#### 3.2.1 Promoting gene expression

Following activation, YAP/TAZ enters the nucleus to bind TEAD and the YAP/TAZ-TEAD complex is directly recruited to promoter regions. Enhancer-bound YAP/TAZ–TEAD complexes regulate gene transcription by inducing p300-dependent 114, through recruitment of the mediator complex and induction of transcriptional elongation through the RNA polymerase II pause release (25). In this way, YAP/TAZ controls the expression of genes directly involved in the cell cycle, anti-apoptotic genes of the B cell lymphoma-2 (Bcl2) and inhibitor of apoptosis (IAP) families and metabolic gene (26). YAP/TAZ may abnormally regulate cell cycle transcription factor AP-1 inducing over-proliferation, cellular migration and metastasis. In numerous tumor cell lines, the YAP/TAZ-TEAD complex mediated AP-1interaction with regulatory regions of the genome affecting transcriptional programs relevant to cell cycle regulation (27). Moreover, in nutrient-limited situations, hyperactive YAP/TAZ can promote the metabolic switch to aerobic glycolysis up-regulating the translocation of glucose and high-affinity amino acids (28).

#### 3.2.2 Increasing metabolism

YAP/TAZ can increase metabolism to supply energy and nutrients to cancer cells. For example, YAP interacts with the carbohydrate response element binding protein in the nucleus of the hepatocytes thereby promoting glycolysis and lipogenesis (29). In metastatic colorectal cancer cells, the glycolytic gene Glut3 appears upregulated, and Glut3 promoted the invasiveness and stemness of tumor cells by activating YAP. Activation of YAP in turn increases Glut3 expression and enhanced glycolysis, promoting tumor growth (30). Increased glutamine catabolism is another important feature of tumor metabolism, providing tumor cells not only with energy but also with nitrogen required for the synthesis of proteins, nucleotides, and lipid macromolecules. Activation of YAP/TAZ also upregulates the expression and activity of glutamine synthetase and glutamine levels, and enhances the nucleotide *de novo* synthesis pathway, thereby promoting tumor cell growth (31).

#### 3.2.3 Maintaining stemness

The upregulation of tumor stem cells and EMT maintains tumor cell stemness and leads to chemoresistance. In ovarian cancer, myosin phosphatase targeting proteins 1 (MYPT1) can activate the Hippo pathway, which normally suppresses YAP-dependent target gene expression and prevents stemness. However, downregulation of MYPT1 leads to Hippo pathway inactivation, thereby allowing YAP-dependent target gene expression increasing cell proliferation, dedifferentiation to a cancer-stem-cell-like state and resistance to platinum-based therapies (32). TAZ is also involved in cancer stem cell self-renewal. TAZ forms a complex with the cell polarity determining cluster Scribble in epithelial cells; TAZ expression is suppressed when Scribble is localized to the membrane. As the tumor progresses, cells lose polarity as they shift from epithelial to mesenchymal, and Scribble is lost in translocation, thereby activating TAZ, in turn enhancing EMT. TAZ was also shown to induce EMT and EMT enhanced TAZ expression. Low Scribble expression in was associated with poor prognosis, which was associated with EMT caused by TAZ overexpression (33).

### 3.3 Hippo pathway and tumor microenvironment

The tumor microenvironment includes cancer, immune, stromal, and endothelial cells, cancer-associated fibroblasts, as well as inflammatory and growth factors, chemokines, etc. The Hippo pathway regulates the tumor microenvironment by inducing the release of inflammatory factors and chemokines, which recruit immune cells that suppress tumor growth while impairing the activity of anti-tumor immune cells.

Upregulation of YAP in EC cells increased the expression of interleukin (IL) -6 and IL-11, facilitating EC growth. By contrast IL-6 and IL-11 expression in tumor cells was decreased by suppression of YAP (34). In triple-negative breast cancer, Hippo mediated the proliferation and migration of tumor-associated macrophages (TAMs) by upregulating IL-34 expression. Meanwhile, Hippo/YAP upregulated the expression of the immune checkpoint molecule programmed death ligand-1 (PD-L1) to inhibit T-cell infiltration, leading to tumor cell recurrence and metastasis (35). In a mouse model of prostate cancer, YAP-TEAD induced expression of IL-6, colony-stimulating factors1-3, tumor necrosis factor-α, IL-3, C-X-C motif chemokine ligands1/2/5 and C-C motif chemokine ligand 2, and recruited myeloid-derived suppressor cells into the tumor microenvironment. These cells secrete a variety of cytokines, chemokines, and enzymes that inhibit the activation and function of T cells and tumor-promoting effects by suppressing host immune response and promoting immune tolerance (36). In addition, YAP/TAZ has the ability to regulate T cell immunity directly. In melanoma, lack of YAP increases CD4^+^T lymphocyte/Treg and CD8^+^T lymphocyte/Treg ratios, but overexpression of YAP inhibits CD8^+^ T cells’ function to produce cytotoxicity and eliminate tumors (37).

### 3.4 Hippo pathway and tumor drug resistance

High nuclear expression of YAP/TAZ-TEAD in the nucleus is associated with drug resistance and poor prognosis in multiple cancers. Overactivation of YAP/TAZ is established as one of the most important mechanisms of drug resistance.

Chemotherapy resistance is associated with dysregulation of various upstream YAP/TAZ regulators, most notably miRNAs. Multiple miRNAs are up-or down-regulated in response to chemotherapy drugs inducing chemo-resistance by targeting different components of the Hippo pathway. Chemotherapy activates activated cyclin-dependent kinase 1 (CDK1), which phosphorylates and promotes LATS binding to and inhibition of Peptidyl-prolyl isomerase, an oncoprotein that induces chemo resistance (38). Over-activation of YAP enhances tumor cell stemness, leading to tumor resistance to cisplatin. Furthermore, YAP/TAZ may also indirectly promote taxane resistance by upregulating the anti-apoptotic proteins Bcl-xL, cIAP1 and Survivin while suppressing protein 53 (p53) (12). YAP/TAZ also induces resistance to multiple targeted therapies, particularly against epidermal growth factor receptor (EGFR) and MAPK inhibitors. Overexpression of YAP promotes transcription of cell cycle-related gene expression, EMT, and cell apoptosis evasion genes, thereby increasing drug resistance (12, 39, 40). As regards immunotherapy, YAP/TAZ-TEAD can directly target the PD-L1 gene and enhance its promoter to increase expression while inhibiting activity of CD8^+^ T lymphocyte, thus enabling immune escape of tumor cells (12, 41) (**Table 1**).

**Table 1:** Effect of the Hippo pathway on cancers.

Component of Hippo	Effect	Mechanism	Cancer type	Refs
YAP	Upregulates genes about proliferation	Modulates Pol II	Liver cancer	(25)
YAP/TAZ	Activates genes about mitosis	Co-occupy chromatin with AP-1	Breast cancer	(27)
YAP	Upregulates cancer metabolism	Enhances the expression of Glut3	Colon cancer	(30)
YAP	Increases the stemness		Ovarian cancer	(32)
TAZ	Increases the stemness		Breast cancer	(33)
YAP	Regulates the tumor microenvironment	Upregulates inflammatory factors and chemokines	Endometrial cancer	(34)
			Breast cancer	(35)
			Prostate cancer	(36)
		Upregulates PD-L1	Breast cancer	(35)
			Melanoma	(37)
YAP	Induces chemotherapy resistance		Ovarian cancer	(32)
			Cervical cancer and Lung cancer	(38)
YAP	Induces targeted therapies resistance	Upregulates EGFR	Esophageal cancer	(39)
YAP	Induces immunotherapy resistance	Upregulates PD-L1	Breast cancer	(35)
			Melanoma	(37)

Pol II, polymerase II; AP-1, Activator Protein-1; PD-L1, programmed death ligand-1.

## 4 Hippo pathway and EC

### 4.1 YAP/TAZ is highly expressed in EC

The level of YAP/TAZ was raised in EC, and expression of YAP was higher in type 2 EC (oestrogen-independent) compared to type 1 (oestrogen-dependent) EC cells (12, 42–44). Nuclear YAP expression in type 1 EC cells was significantly associated with poorer overall survival, whereas there was no correlation between YAP expression and overall survival in type 2 EC (12). Interestingly, cellular expression of YAP was considerably lower in the patients treated by metformin which has been proposed to have antineoplastic properties against EC (45). A study detected expression of TAZ in different types of EC evaluated by immunohistochemistry. TAZ expression was detected in 76% of undifferentiated EC, in 54% of endometrial carcinosarcomas, in 46% of endometrial serous carcinomas, in 36% of grade 3 EC, and in 18% of grade 1-2 EC. The same trend was observed in cellular studies: TAZ was significantly more expressed in hyperdifferentiated AN3CA cells, while well-differentiated Ishikawa cells expressed TAZ relatively poorly (46). These findings demonstrate that TAZ was expressed to a greater extent when EC were more poorly differentiated.

### 4.2 Hippo pathway affects biological behavior of EC

The proliferation, migration, and invasion of EC cells were decreased after YAP was downregulated and elevated when YAP was overexpressed, as demonstrated by Cheng et al. Additionally, activation of the Hippo pathway was shown to suppress the stemness of EC, while loss of Hippo pathway signaling promoted the maintenance and expansion of CSCs (47). Calmodulin and E-cadherin in EC cells rose after YAP inhibition, and these trends were reversed after YAP overexpression, proving that Hippo was engaged in the EMT process in EC and then maintained the stemness of tumor cells (12). YAP also increased the transcription of cyclin D1 which stimulates the aggressiveness of EC cells (43, 48). Research has shown that TAZ overexpression induced cell motility, invasiveness, and tumor growth in early EC cells. Upregulation of TAZ promoted the expression of the EMT inducers, and was associated with loss of E-cadherin, while TAZ silencing decreased vimentin expression. A similar trend has also been identified in human tumors (46).

### 4.3 Hippo regulators and EC

Over 90% of EC patients show aberrant PI3K/Akt signaling, which, as described above, leads to activation of YAP/TAZ (49). YAP1 has also been found, in turn, to activate the PI3K/Akt pathway regulating EC cell proliferation. Furthermore, YAP1 has been found to activate the PI3K pathway through upregulation of growth factor binding protein 2 associated binding protein 2 (GAB2) linker molecules. In addition, siYAP/TAZ suppressed cell proliferation more potently than YAP or TAZ knockdown alone (50). p190A is a highly efficient GTPase-activating protein involved in cell migration, polarity, and cell cycle processes through regulation of cytoskeleton and cell adhesion dynamics. In fact, p190A is an upstream regulator of the Hippo-YAP pathway and has been found to be downregulated in about 20% of EC patients. Knockdown of the p190A gene resulted in YAP activation, which in turn promoted the proliferation and migration of EC cells (42). When FAT tumor suppressor homologue 4 and lipolysis-stimulated lipoprotein receptors, essential components of cell contacts and junctions, were silenced, there was a reduction in the phosphorylation YAP and an increase in YAP nuclear translocation, which stimulated EC proliferation and invasion (51). In addition, several ncRNAs can also regulate the Hippo pathway in EC. MiRNA 31 promoted translocation of YAP1 into the nucleus and promotes the transcription of cyclin D1 in EC cells (48). The expression of lncRNA FRMD6-AS2 was found to be reduced in EC patients, and upregulation of FRMD6-AS2 increased phosphorylation of YAP, which prevented EC cells from migrating and invading (52). These regulators of Hippo may be the potential target in EC.

## 5 Hippo pathway as therapeutic target in EC

Given that there are numerous upstream activators susceptible to oncogenic bypass and potential oncogenic proteins. Targeting Hippo components directly seems to be a feasible strategy to regulate the Hippo pathway It has been shown that the selective MST1/2 inhibitor XMU-MP-1 stimulates YAP activation, which in turn promotes cell proliferation and tissue repair and regeneration (53), but its anti-tumor effect needs further study. Currently, inhibitors that target YAP/TAZ-TEAD are being studied in preclinical and clinical trials with promising outcomes. Hippo has additionally been shown to be a potential target in some anticancer medications approved by the Food and Drug Administration (FDA).In EC and other malignancies, drugs targeting the Hippo pathway are summarized in **Table 2**.

**Table 2:** Drugs targeting the Hippo pathway.

Name	Mechanism	Potential Hippo target	Cancer type of research or FDA indication	Clinical trials or FDA approval	Refs
XMU-MP-1	Inhibits MST1/2 selectively	MST1/2			(53)
VP	Increases 14-3-3σ	YAP-TEAD	EC		(44, 50)
	Increases photosensation		Primary Breast cancer	Phase I/II	(54)
	Sensitizes cancer cells to the chemotherapy	YAP	Colorectal cancer		(55)
Lenvatinib	Reduces expression and increases the phosphorylation of YAP	YAP	Advanced EC after progression or following prior systemic therapy	Approved	(56)
Pazopanib	Inhibits the nuclear localization of YAP/TAZ	YAP/TAZ	Recurrent or metastatic uterine sarcoma following prior cytotoxic therapy	Approved	(57)
Metformin		YAP	Stage I EC	Phase III	(58)
	Sensitizes cancer cells to the chemotherapy	YAP	Advanced and recurrent EC	phase II/III, with paclitaxel and carboplatin,	(59)
		YAP	Advanced and recurrent EC	Phase II, with Everolimus and Letrozole	(60)
	downregulates IGF1R and PI3K-AKT-mTor pathways	YAP	Advanced and recurrent EC	Phase I/II, with Cyclophosphamide and Olaparib	(61)
Dipeptidyl peptidase‐IV inhibitors	Inactive YAP in a LATS homolog-dependent	YAP	Gastric cancer		(62)
Statins	Inactivate TAZ to activate p53		Osteosarcoma		(63)
	Inhibit YAP to Reverse primary resistance to EGFR inhibitors		Colorectal cancer		(64)
VT3989	Binds central pocket of TEAD	TEAD	Malignant pleural mesothelioma and solid tumors with NF2 mutation	Phase I	(65)
IK-930	Binds central pocket of TEAD	TEAD	Malignant pleural mesothelioma and solid tumors with NF2 mutation	Phase I	(66)
IAG933	Binds surface of TEAD	TEAD	Malignant pleural mesothelioma and solid tumors with NF2 mutation	Phase I	(67)
GSK3 inhibitors	Prevent YAP/TAZ proteasomal degradation	YAP/TAZ	Pancreatic Cancer EC		(68, 69)

VP, Verteporfin; NF2, neurofibromatosis type 2; GSK3, Glycogen synthase kinase 3.

### 5.1 Verteporfin

**Figure 2:**
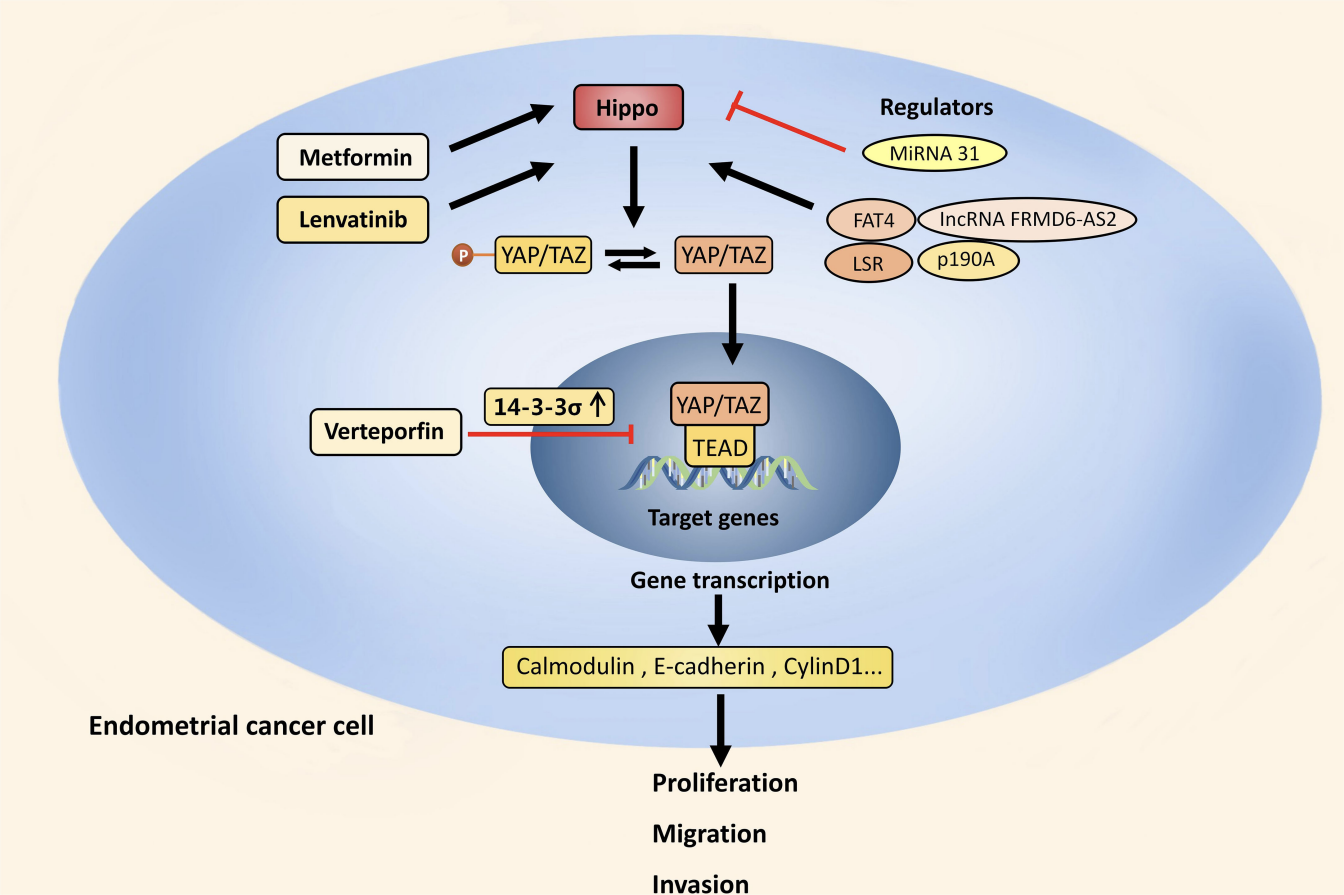
Hippo pathway and EC. In EC, the Hippo pathway is suppressed and activated YAP/TAZ increases the transcription of calmodulin, E-cadherin and cyclin D1. This stimulates the EMT process and maintains stemness, which in turn cause the proliferation, migration, and invasion of EC cells. The Hippo pathway can be upregulated by PI3K/Akt signaling, p190A, FAT4, LSR and lncRNA FRMD6-AS2, and downregulated by MiRNA 31. Through increasing 14-3-3σ, VP prevents EC from developing by retaining YAP in the cytoplasm. FDA approved drugs like Metformin and Lenvatinib also have anti-tumor effect by upregulating the Hippo pathway.

VP, a second-generation photosensitizer approved for treatage-related macular degeneration, was utilized for photodynamic therapy in a phase I/II study recruiting patients diagnosed with primary breast cancer (54). By inhibiting YAP-TEAD binding, VP can also impede the proliferation of tumor cells. It was identified to enhance sensitivity and lower resistance to anticancer therapy by reducing YAP expression (70). Shi has demonstrated that YAP overexpression increased the sensitivity of cancer cells resistant to paclitaxel, and this was reversed by treatment with VP (55). In EC, VP had the effect of lowering YAP protein levels and dramatically reducing the number and size of tumors *in vivo*. Experimentally, three control mice showed tumors extending into the abdominal cavity with abdominal wall adhesions, whereas none of the mice treated with VP showed peritoneal spread (50). In terms of the mechanism, VP retained YAP in the cytoplasm by increasing the amount of 14-3-3σ, a YAP protein regulated by p53 (44). Therefore, cells with wild-type p53 may show the effects of VP evidently (**Figure 2**).

### 5.2 Tyrosine kinase inhibitors

Lenvatinib is an oral multitarget TKI and currently approved with pembrolizumab for advanced EC after progression or following prior systemic therapy (56). Study showed that Lenvatinib reduced YAP expression and increased the phosphorylation of YAP at both Ser127 and Ser397 both *in vivo* and *in vitro*. XMU-MP-1 mediated YAP activation and overexpression effectively attenuated the Lenvatinib-induced decrease (71). Cabozantinib is approved for renal cell carcinoma, medullary thyroid cancer, and hepatocellular carcinoma, and currently being tested in a phase II clinical trial with nivolumab for the treatment of patients with advanced or metastatic EC (72). The transcription factor sex-determining region on Y box 17 (SOX17) serves as a tumor suppressor and is frequently mutated in EC, which can bind to TEAD transcription factors and decrease the DNA-binding ability of TEAD, and restrict the proliferation, migration and invasion of EC cells consequently. Cabozantinib which inhibits the YAP/TEAD transcriptional target AXL can reverse malignant transformation of EC cells caused by SOX17 loss (73) (**Figure 2**). Further study is required to determine whether Cabozantinib inhibits EC directly through the Hippo. Pazopanib is another TKI approved for soft tissue sarcoma after one prior chemotherapy regimen. It is currently indicated for patients with recurrent or metastatic uterine sarcoma following prior cytotoxic therapy (57). It was demonstrated that YAP/TAZ transcriptional co-activators can be inhibited by Pazopanib, especially in YAP/TAZ-dependent cancer cells (74). Therefore, expression or activation status of YAP/TAZ may be a predictor for Pazopanib.

### 5.3 Medications for metabolic diseases

Some drugs for diabetes and cardiovascular diseases have also shown anti-cancer potential and the Hippo pathway may be a potential target. These metabolic diseases medications have been approved for clinical practice, have good bioavailability, and are almost not harmful to healthy cells or tissues, making it worthwhile to explore their therapeutic potential in EC. Metformin is a first-line oral hypoglycemic drug and functions by increasing insulin sensitivity in peripheral tissues. Apart from its antidiabetic effect, it has been suggested in fertility-sparing treatment for early EC in addition to progestin therapy and proved to reduce mortality and prevent recurrence of EC (58, 75, 76). In EC clinical trials, Metformin was used with paclitaxel+carboplatin (59), Everolimus+Letrozole (60), and Cyclophosphamide+Olaparib (61). One of the mechanisms behind its anti-cancer action in EC is upregulating the Hippo pathway (45) (**Figure 2**). Dipeptidyl peptidase‐IV inhibitors are emerging kind of oral hypoglycemic agent which reduce the proliferation of cancer cells by inactivating YAP in a LATS homolog-dependent way, and this inhibition can be blocked by AMPK (62). Statins, which are prescribed to lower cholesterol, was verified to inhibit TAZ to activate p53 by reducing geranylgeranyl diphosphate (77), thereby inhibiting cancer cell growth (63). Statins can also inhibit YAP, reversing primary resistance to EGFR inhibitors as a result (64).

In EC, the Hippo pathway is suppressed and activated YAP/TAZ increases the transcription of calmodulin, E-cadherin and cyclin D1. This stimulates the EMT process and maintains stemness, which in turn cause the proliferation, migration, and invasion of EC cells. The Hippo pathway can be upregulated by PI3K/Akt signaling, p190A, FAT4, LSR and lncRNA FRMD6-AS2, and downregulated by MiRNA 31. Through increasing 14-3-3σ, VP prevents EC from developing by retaining YAP in the cytoplasm. FDA approved drugs like Metformin and Lenvatinib also have anti-tumor effect by upregulating the Hippo pathway.

### 5.4 TEAD inhibitors

To upregulate various transcriptional programs that promote cancer development, YAP/TAZ need to complex with TEAD transcription factors. Therefore, the structure of TEAD complexed with YAP/TAZ making it a potential therapeutic target. Generally, patented TEAD inhibitors mainly target central and surface hydrophobic pockets of TEAD (78). Three TEAD-inhibiting small molecules are currently being tested in Phase I clinical trials: VT3989-NCT04665206 (binding central pocket) (65), IK-930-NCT05228015 (binding central pocket) (66), and IAG933-NCT04857372 (binding surface) (67). VT3989 and IK-930 have been tested in clinical trials for monotherapy and also in combination with Osimertinib or Trametinib in preclinical studies (78–80).

### 5.5 Glycogen synthase kinase 3 inhibitor

GSK3 is a multifunctional serine/threonine kinase that has been implicated in a number of human malignancies. By preventing YAP/TAZ proteasomal degradation, GSK3 inhibitor is able to reduce YAP/TAZ and the transcription of YAP/TAZ-regulated genes, therefore suppress their oncogenic functions (68). Although the role of GSK3 in EC has not yet been fully determined, research has shown that inhibiting GSK3 suppressed EC growth and proliferation and made EC cells more sensitive to Paclitaxel (69).

## 6 Concluding remark and future direction

The core component of the Hippo pathway is MST1/2-LATS1/2-YAP/TAZ-TEAD, which is regulated by multiple upstream signals. Inhibiting the Hippo pathway causes activation of its primary effector, YAP/TAZ, which modulates the tumor microenvironment and triggers pro-oncogenic effects. In EC, multiple upstream signals influence how YAP/TAZ affects tumor invasion, migration, and proliferation. Drugs targeting Hippo for monotherapy in EC may not be as effective as in those tumors with frequent NF2 deficient. Considering that Hippo-YAP/TAZ activation is established as a major factor of resistance to anti-tumor medications, traditional antitumor therapies combined with therapies targeting the Hippo pathway may be a trend in the coming years and ultimately benefit EC patients. We hope the review to provide fresh perspectives to explore new mechanisms and potential therapeutic target of EC.

## Author contributions

XS: Writing – original draft. QL: Writing – review & editing. YS: Writing – review & editing. LC: Writing – review & editing. FX: Writing – review & editing. WT: Writing – review & editing. YW: Writing – original draft.
